# Immune activation and arterial stiffness in lean adults with HIV on antiretroviral therapy

**DOI:** 10.4102/sajhivmed.v22i1.1190

**Published:** 2021-03-19

**Authors:** Longa Kaluba, Fastone Goma, Chris Guure, Sody Munsaka, Wilbroad Mutale, Douglas C. Heimburger, Theresa Chikopela, John R. Koethe

**Affiliations:** 1 School of Medicine, Cavendish University Zambia, Lusaka, Zambia; 2 Eden University, Lusaka, Zambia; 3 Department of Physiological Sciences, School of Medicine, University of Zambia, Lusaka, Zambia; 4 Department of Biostatistics, School of Public Health, University of Ghana, Legon, Ghana; 5 Department of Biomedical Sciences, School of Health Sciences, University of Zambia, Lusaka, Zambia; 6 Department of Health Policy and Management, School of Public Health, University of Zambia, Lusaka, Zambia; 7 Vanderbilt Institute for Global Health and Department of Medicine, Vanderbilt University Medical Centre, Nashville, TN, United States of America; 8 Department of Human Physiology, Faculty of Medicine, Lusaka Apex University, Lusaka, Zambia

**Keywords:** endothelial dysfunction, immune activation, lean adults, antiretroviral therapy, arterial stiffness

## Abstract

**Background:**

Greater T-cell activation was associated with reduced vascular compliance amongst persons living with HIV (PLWH) especially among overweight and obese individuals. There is a paucity of data regarding immune activation and arterial stiffness amongst PLWH in sub-Saharan Africa (SSA).

**Objective:**

To determine the association between immune activation and arterial stiffness in lean PLWH in SSA.

**Method:**

Forty-eight human immunodeficiency virus positive (HIV+) adults on antiretroviral therapy (ART) >5 years and 26 HIV-negative adults, all with BMI < 25 kg/m^2^ and no history of CVD, were enrolled. The relationship of vascular compliance with circulating CD4+ and CD8+ naïve, memory, activated and senescent T cells, and serum 8-isoprostane was assessed by HIV status.

**Results:**

Increased immune activation was observed in the CD4+ and CD8+ T cells of PLWH, 16.7% vs. 8.9% and 22.0% vs. 12.4% respectively; *p* < 0.001 (both). Furthermore, a higher proportion of senescent CD4+ T cells were associated with a lower carotid-femoral pulse wave velocity (cfPWV; *p* = 0.01), whilst a higher proportion of activated CD8+ T cells were associated with a lower carotid-radial pulse wave velocity (crPWV; *p* = 0.04), after adjustment for BMI and age. However, PLWH also had a higher median carotid-femoral augmentation index (cfAiX) (21.1% vs. 6.0%; *p* < 0.05) in comparison to their HIV controls.

**Conclusion:**

Our population of lean PLWH had increased immune activation and higher cfAiX, a marker of arterial stiffness, compared to HIV-negative persons. The negative association between immune activation and arterial stiffness as measured by crPWV in PLHW on long-term treatment needs further elucidation.

## Introduction

The prevalence of non-communicable diseases in sub-Saharan Africa (SSA) has continued to rise over the last two decades^1,2^ with cardiovascular-related disorders being a leading cause of death in persons living with HIV (PLWH).^3^ Endothelial dysfunction, a proposed mechanism leading to this increased cardiovascular disease (CVD ) risk, results in diminished relaxation of the vascular smooth muscle and to arterial stiffness.^4,5,6^

In the general population, a meta-analysis^7^ found that an increase of 1 metre per second (m/s) in pulse wave velocity (PWV), a marker of greater arterial stiffness, was associated with a 14% increased risk of cardiovascular events and 15% increased risk of all-cause mortality when adjusted for age, sex and cardiovascular risk factors. Increased arterial stiffness and immune activation have been reported in PLWH.^8,9^

Persons living with human immunodeficiency virus (HIV) have reduced levels of endothelial nitric oxide synthase (eNOS), an important regulator of vascular compliance.^10^ Further, other factors such as immune activation, inflammation, oxidative stress and HIV proteins have also been associated with impaired vascular compliance in PLWH.

Immune activation is increased in PLWH compared to HIV-negative persons.^6,11^ Recent studies, predominantly from cohorts in the United States (US), report associations between circulating T-cell subsets and vascular compliance in PLWH. Kaplan, Sinclair^12^ reported a positive association between activated CD4+ T cells and arterial stiffness after adjusting for HIV ribonucleic acid (RNA) and total peripheral CD4+ T-cell count. A longitudinal study by Karim, Mack^9^ also reported a positive correlation between activated CD4+ T cells and carotid arterial stiffness both pre- and post-highly active antiretroviral therapy (HAART) initiation. CD8+ T-cell activation was associated with reduced smooth muscle relaxation independent of other cardiovascular risk factors in PLWH with viral suppression.^13,14^

Whilst the mechanistic link between T-cell activation and vascular compliance is not fully understood, immune cells are capable of generating reactive oxygen species and contributing to oxidative stress.^15^ Higher levels of reactive oxygen species and circulating pro-inflammatory cytokines, two conditions commonly increased in PLWH despite effective plasma viral suppression on antiretroviral therapy (ART), contribute to endothelial dysfunction and reduced vascular compliance.^16,17,18,19,20^ Higher 8-isoprostane, a marker of oxidative stress, is associated with subsequent all-cause mortality in PLWH independent of CD4+ T-cell count and high-sensitivity C-reactive protein (hsCRP).^21^ The overproduction of reactive oxygen species such as superoxide, which is formed when oxygen is reduced by a single electron, has been associated with the progression of cardiovascular disorders.^18^ Increased superoxide binds to the increased nitric oxide (NO) to form peroxynitrite, which results in the production of a powerful oxidant that traverses into the endothelial cell and directly damages the deoxyribonucleic acid (DNA); compromising the bioavailability of NO and resulting in reduced relaxation.^16,18^ In addition, HIV proteins have also been implicated in endothelial dysfunction.^22^

In SSA, the burden of CVD in PLWH has tripled over the last two decades.^23^ However, much of our current understanding of HIV and cardiovascular function arises from cohort studies in the US and Europe, which often include a high proportion of overweight and obese individuals reflective of the general population.^24^ Whilst rates of obesity are rising in many SSA countries, particularly South Africa, there remains a broad distribution of body composition amongst PLWH in the region which includes a substantial number of individuals with lower body mass index (BMI) values. At present, there is a paucity of data on whether inflammation and immune activation in ‘lean’ (i.e. BMI < 25 kilogram per square metre [kg/m^2^]) PLWH is accompanied by a similar degree of arterial stiffness as observed in higher BMI individuals. To this end, we assessed relationships between cellular immune activation, oxidative stress and arterial stiffness in a cohort of lean PLWH on long-term ART.

## Materials and methods

We conducted an analytical cross-sectional study at University Teaching Hospital (UTH) in Lusaka, Zambia between September 2018 and June 2019 amongst PLWH on long-term ART with BMI’s <25 kg/m^2^. Persons accompanying patients to the ART clinic and general medicine outpatients department were recruited as HIV-negative controls. The HIV infection status was confirmed in all HIV-negative participants by rapid test. All PLWH were on a regimen of efavirenz, emtricitabine, and tenofovir disoproxil fumarate for more than 5 years. Persons who were pregnant, or with known CVD, rheumatologic disease or any other self-reported pathology, active infectious conditions aside from HIV, or with a history of diabetes and habit of tobacco smoking were excluded. Socio-demographic data were collected using the WHO STEPS questionnaire.^25^ Randomly sampled participants provided written informed consent, and ethical approval was obtained.

## Endothelial dysfunction measurements

Carotid-femoral pulse wave velocity (cfPWV) and carotid-radial pulse wave velocity (crPWV) are measures of arterial stiffness, calculated from the time taken for the arterial pulse to propagate from the carotid to the femoral or radial artery, respectively. Carotid-femoral pulse wave velocity is said to be the gold standard in the measure of aortic stiffness.^26^ Other derivatives of PWV measurement include augmentation index (AiX) which is the measure of the enhancement of the central aortic artery reflective wave and is said to be a more sensitive marker for vascular compliance.^27^ All these parameters were measured using the ALAM Complior Analyse device (ALAM medical, France). Non-invasive probes were applied to the surface of the skin over the carotid, femoral and radial arteries with participants lying in a supine position, after resting for a minimum of 5 min. Participants were not allowed to move, speak, or sleep during the measurements.^28^

## Biochemical measurements

Fasting blood was collected in an ethylenediamine tetraacetic acid (EDTA) tube and analysed by flow cytometry. A lyse-wash protocol (Becton Dickinson) was used to stain lymphocytes. A 100 microlitres (µL) of whole blood was stained with the following monoclonal antibodies: CD3 APC (Sigma Aldrich, clone MEM-57), CD4 PerCP (Thermofisher, clone RM4-5), CD8 APC-Cy7 (Biolegend, clone HIT8a), HLA DR PE-Cy7 (Thermofisher, clone L243), CD57 PE (Thermofisher, CD57, clone TB01), CD45RA FITC (Sigma Aldrich, clone MEM-56). Fluorescence-activated cell sorting (FACS) lysing solution was added, followed by centrifugation to isolate the WBCs.^29^

T-cell subsets were identified using sequential gating on a FACSverse flow cytometer using FACSuite software (Becton Dickinson, San Diego, CA). Lymphocytes were gated using forward, and side scatter (forward scatter [FSC] and side scatter [SSC]) (Appendix Figure 1).

The cells were then gated by the expression of fluorochromes. To overcome the physical overlap of the emission spectra of common fluorochromes, unstained and single stained controls, and fluorescence minus one (FMO) controls were used as a reference to automatically generate compensation matrices using the Flowjo version 10 software. These matrices were manually verified.

## Oxidative stress measurements

Serum samples stored at −80 °C were thawed for measurement of 8-isoprostane. An enzyme-linked immunosorbent assay (ELISA) was performed using an immobilised monoclonal antibody of 8-isoprostane (Detroit R&D, Cat # 8iso1) as specified by the manufacturer’s instructions.^30^

## Statistical analyses

Because of inconsistent distribution of outcome variables, medians and interquartile ranges were calculated for continuous variables and percentages for categorical variables. The relationships between clinical and demographic characteristics were assessed using Mann-Whitney U test or chi-squared test as appropriate. We assessed whether T-cell subsets were associated with markers of endothelial function (crPWV, cfPWV, carotid-radial augmentation index [crAiX], carotid-femoral augmentation index [cfAiX]) and oxidative stress (8-isoprostane) using multivariate models adjusted for age and BMI in the PLWH. The normality of the outcome data was assessed using Q-Q plots and validated with the Shapiro–Wilk test. Only crPWV required log-transformation.

Multiple linear regression models were used to assess relationships between the independent variables (CD4+ and CD8+ T-cell subsets) and the dependent variables (PWV, AiX and 8-isoprostane); these models were adjusted for BMI and age in the HIV+ group only, because of the small sample size.

Linear regression models incorporating an interaction between CD4+ and CD8+ T-cell subsets with HIV status, adjusted for BMI and age, were also performed (Appendix Table 1-A1). The results of regression models are reported using Beta (β) coefficients, confidence intervals (CI) and *p*-values. Analyses were performed using STATA version 15 software.

### Ethical considerations

Participants provided written informed consent, and approval was obtained from the University of Zambia Biomedical Research Ethics Committee (UNZABREC reference number 003-01-18) and the National Health Research Authority (NHRA).

## Results

A total of 74 adult participants were enrolled in the study: 48 with HIV (28 female; 20 male) and 26 HIV-negative (15 female; 11 male). The median age of the PLWH was 41 years versus 23 years amongst the HIV-negative group (Table 1). People living with HIV had a lower BMI compared to the HIV-negative group (18.9 kg/m^2^ vs. 20.7 kg/m^2^, respectively) but had comparable waist circumferences (72 centimetres [cm] vs. 70 cm). Median central blood pressure measurements were also comparable between groups (112/78 vs. 116/77). Both cfPWV and crPWV fell within normal ranges of 9.1 m/s ± 3.2 m/s^31,32^ (Table 2). Carotid-femoral augmentation index was significantly higher in PLWH (Table 2). Other measurements of endothelial function and 8-isoprostane were similar between groups.

**TABLE 1:** Clinical and demographic characteristics.

Variable	PLWH (*n* = 48)					HIV-negative (*n* = 26)					*P*
	Mdn	IQR	%	SD	Mean	Mdn	IQR	%	SD	Mean	
Age (years)	41	36, 43.5	-	-	-	22.5	20, 27	-	-	-	**< 0.001**
Female (%)	28	-	58	-	-	15	-	58	-	-	0.96
Height (cm)	164.5	157.5, 171	-	-	-	166.5	160, 173	-	-	-	0.38
Weight (kg)	51.7	47.9, 55.2	-	-	-	55.2	52.8, 61.6	-	-	-	**0.01**
BMI	18.9	17.4, 20.6	-	-	-	20.7	19.1, 22.9	-	-	-	**< 0.01**
Waist (cm)	72	67.2, 74.1	-	-	-	70	66.3, 73	-	-	-	0.81
Hip (cm)	86.4	83.5, 92.8	-	-	-	89.3	86.5, 97.8	-	-	-	0.06
Heart rate (beats/min)	67	61, 74	-	-	-	67	59, 77	-	-	-	0.62
Central systolic blood pressure (mmHg)	-	-	-	± 19	112.4	-	-	-	± 19.6	116.1	0.27
Central diastolic blood pressure (mmHg)	-	-	-	± 10.3	78.2	-	-	-	± 7.8	77.4	0.99
Body fat (%)	18.5	12.3, 26.2	-	-	-	18.6	9.7, 26.1	-	-	-	0.74
Fat mass (kg)	9.5	6, 12.6	-	-	-	11.2	4.9, 14.3	-	-	-	0.67
Trunk fat (%)	16.7	8.3, 21.8	-	-	-	15.7	8.4, 21.5	-	-	-	0.77
Trunk fat mass (kg)	4.7	2.8, 6.5	-	-	-	4.3	2.5, 6.7	-	-	-	0.93
Total trunk free fat mass (kg)	23.4	21.1, 26.1	-	-	-	25.7	23.7, 27.4	-	-	-	**0.01**

Note: Medians and interquartile ranges. Body composition measurements utilised body impedance analysis (BIA) by the Tanita BC418 MA. Mann-Whitney U test or chi-squared test was used. *p*-values < 0.05 are shown in bold.

HIV, human immunodeficiency virus; PLWH, persons living with HIV; BMI, body mass index.

**TABLE 2:** Pulse wave velocity and oxidative stress indices.

Variable	PLWH (*n* = 48)		HIV-negative (*n* = 26)		*P*
	Median	IQR	Median	IQR	
**Pulse wave velocity indices**					
cfPWV (m/s)	7.3	6.1, 8.8	7.0	5.8, 7.7	0.15
crPWV (m/s)	9.9	8.8, 10.8	8.7	7.3, 10.8	0.09
cfAiX	21.1	5.9, 35.6	6.0	−5.7, 24.5	**0.04**
crAiX	12.9	2.7, 41.4	13.7	−9.7, 26.3	0.20
**Oxidative stress**					
8-isoprostane (pg/µL)	2.6	0.9, 3.7	1.1	0.6, 2.8	0.17

Note: Medians and interquartile ranges. Mann-Whitney U test or chi-squared test was used. *p*-values < 0.05 are shown in bold.

HIV, human immunodeficiency virus; cfAiX, carotid-femoral augmentation index; crAiX, carotid-radial augmentation index; PLWH, persons living with HIV; cfPWV, carotid-femoral pulse wave velocity; crPWV, carotid radial pulse wave velocity.

Naïve CD8+ cells were lower in PLWH compared to the HIV-negative controls (*p* < 0.001). Both activated and memory CD4+ and CD8+ T cells were significantly higher in the PLWH as well (Table 3). Higher activated CD8+ T cells were associated with lower crPWV only in the PLWH (*p* = 0.04). Activated CD4+ T cells were significantly associated with 8-isoprostane (Table 4). A 1% increase in activated CD4+ T cells correlated with a 0.11 picogram/microliter (pg/µL) decrease in 8-isoprostane. Higher senescent CD4+ T cells were associated with lower cfPWV (Table 4). A 1% increase in senescent CD4+ T cells corresponded to a 0.22 m/s decrease in the cfPWV. These relationships were absent or differed in HIV-negative persons (see Appendix Table 2-A2); notably, greater senescent CD4+ T cells was associated with higher cfPWV in this group (a finding contrary to that of participants with HIV).

**TABLE 3:** T-cell subsets.

Variable	PLWH (*n* = 48)		HIV-negative (*n* = 26)		*P*
	Median	IQR	Median	IQR	
**CD4 T-cells (*%*)**					
CD45RA+ (naïve)	20.0	11.1, 28.1	25.3	17.7, 33.2	0.06
CD45RA- (memory)	78	69, 87.6	72.4	64.3, 77.9	**0.04**
HLADR-CD57+ (senescent)	4.4	2.7, 6.5	3.2	2.0, 5.6	0.28
HLADR+CD57- (activated)	15.9	9.2, 21.7	8.4	6.3, 10.9	**< 0.001**
**CD8 T-cells (*%*)**					
CD45RA+ (naïve)	42.6	31.8, 52.2	55.0	49.0, 65.1	**< 0.001**
CD45RA- (memory)	52.7	44.7, 63.7	41.7	29.5, 46.3	**< 0.001**
HLADR+CD57- (activated)	21.55	13.2, 30.2	12.0	6.8, 15.2	**< 0.001**

Note: % total CD4 and CD8 T cells, respectively; Medians and interquartile ranges. Mann-Whitney U test was used. *p*-values < 0.05 are shown in bold.

HIV, human immunodeficiency virus; PLWH, persons living with HIV; CD4, cluster of differentiation 4; CD8, cluster of differentiation 8.

**TABLE 4:** Relationship of T-cell subsets with carotid-femoral pulse wave velocity, carotid-femoral augmentation index, carotid-radial augmentation index, log-transformed carotid-radial pulse wave velocity and 8-isoprostane in persons living with human immunodeficiency virus only.

Outcomes	CD4/CD45RA+ (naïve)			CD4/CD45RA- (memory)			CD4/HLADR-CD57+ (senescent)			CD4/HLADR+CD57- (activated)			CD8/CD45RA+ (naïve)			CD8/CD45RA- (memory)			CD8/HLADR+CD57- (activated)			8- isoprostane		
	β	CI	*P*	β	CI	*P*	β	CI	*P*	β	CI	*P*	β	CI	*P*	β	CI	*P*	β	CI	*P*	β	CI	*P*
cfPWV	−0.01	−0.07–0.07	0.95	−0.01	−0.07–0.07	0.99	−0.22	−0.37–-0.07	0.01	−0.01	−0.11–0.1	0.95	0.05	−0.02–0.12	0.18	−0.04	−0.11–0.03	0.22	−0.07	−0.15–0.01	0.08	−0.13	−0.68–0.43	0.64
cfAiX	0.04	−0.56–0.65	0.88	−0.06	−0.65–0.52	0.82	0.76	−0.74–2.27	0.31	0.20	−0.66–1.07	0.64	−0.01	−0.55–0.52	0.96	−0.01	−0.54–0.51	0.96	0.12	−0.51–0.74	0.71	−0.99	−6.89–4.90	0.73
crPWV	0.01	−0.007–0.009	0.79	−0.01	−0.009–0.007	0.81	−0.01	−0.02–0.01	0.66	−0.01	−0.01–0.01	0.37	0.01	0.002–0.01	**0.01**	−0.01	−0.01–0.002	**0.01**	−0.01	−0.01–0.0002	**0.04**	0.01	−0.05–0.06	0.75
crAiX	0.42	−0.34–1.18	0.27	−0.48	−1.20–0.23	0.17	−0.91	−2.66–0.85	0.29	0.17	−0.79–1.12	0.72	−0.26	−0.89–0.37	0.40	0.27	−0.36–0.89	0.38	0.35	−0.35–1.06	0.31	0.34	−5.72–6.40	0.91
8 isoprostane	0.09	0.05–0.14	**< 0.001**	−0.09	−0.13–0.05	**< 0.001**	−0.01	−0.15–0.12	0.84	−0.11	−17–0.05	**< 0.01**	0.02	−0.04–0.07	0.50	−0.02	−0.07–0.03	0.45	−0.04	−0.10–0.02	0.15	-	-	-

Note: The values of crPWV have been log-transformed. Model adjusted for age and BMI in the PLWH group only. *p*-values < 0.05 are shown in bold.

CI, confidence interval; cfPWV, carotid-femoral pulse wave velocity; crPWV, carotid-radial pulse wave velocity; cfAiX, carotid-femoral augmentation index; crAiX, carotid-radial augmentation index.

Higher memory CD4+ T cells were associated with lower 8-isoprostane concentrations (*p* < 0.001). Each per cent increase in memory CD4+ T cells correlated with a 0.09 pg/µL decrease in 8-isoprostane. Higher memory CD8+ cells were associated with higher crPWV (Table 4; *p* = 0.01). Higher naïve CD8+ cells were associated with higher log-transformed, crPWV (Table 4; *p* = 0.01).

Finally, we performed an interaction analysis given the differing directionality of the relationship between senescent CD4+ T cells and cfPWV in the participants with, versus without, HIV. After adjusting for age and BMI, the relationship of senescent CD4+ T cells with cfPWV differed by HIV status (Figure 1 and Appendix Table 3-A3; interaction term β = −0.22; *p* = 0.05). Similarly, the relationship of senescent CD4+ T cells with carotid-radial augmentation index differed by the HIV status (Figure 2 and Appendix Table 4-A4; interaction term β = −3.46; *p* = 0.03).

**FIGURE 1:**
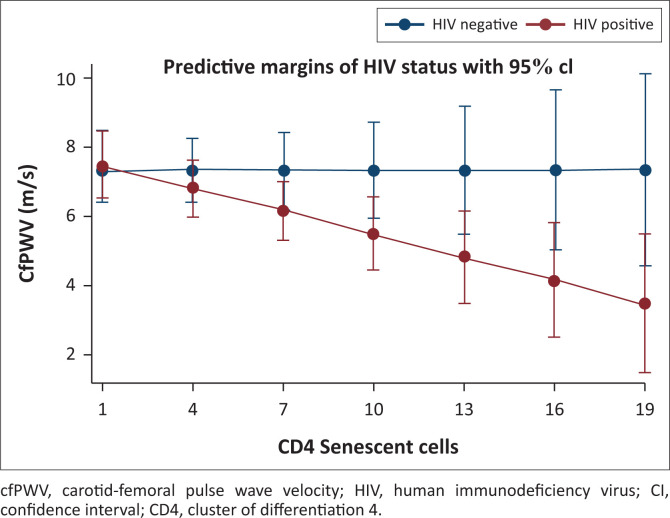
Relationship of carotid-femoral pulse wave velocity and senescent CD4 T cells by human immunodeficiency virus status. Senescent CD4+ cells are shown as the proportion of total CD4+ cells. The model was adjusted for age and body mass index.

**FIGURE 2:**
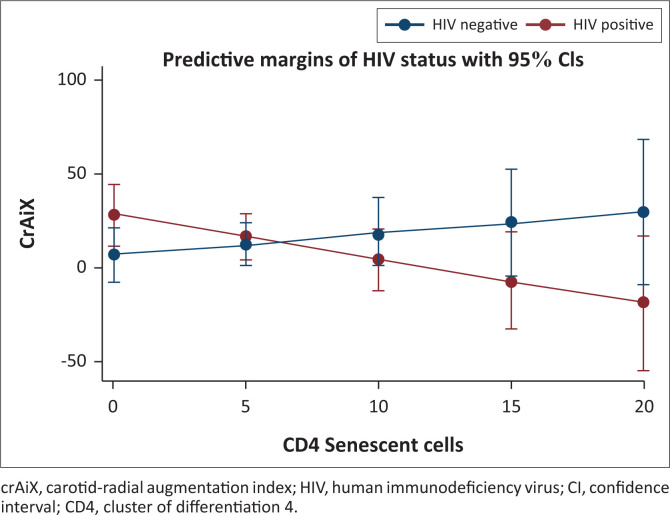
Relationship of carotid-radial augmentation index and senescent CD4 T cells by human immunodeficiency virus status. Senescent CD4+ cells are shown as the proportion of total CD4+ cells. The model was adjusted for age and body mass index.

## Discussion

In the cohort of lean individuals in Lusaka, Zambia, PLWH on long-term ART had greater arterial stiffness (through increased cfAiX), immune activation, and more senescent T cells compared to HIV-negative persons, which is consistent with studies conducted in other settings.^8,9^ However, amongst the lean PLWH in our study higher proportions of senescent CD4+ T cells and activated CD8+ T cells were negatively associated with arterial stiffness as measured by PWV (β = −0.22 and −0.01, respectively). This finding is in contrast to studies in predominantly US cohorts with a high proportion of overweight and obese participants, in which greater T-cell activation (measured by CD38+HLADR+) has been associated with increased arterial stiffness, hypoxia-induced relaxation and increased atherosclerosis.^9,12,13,14^ Furthermore, most of the associations between T-cell subsets and vascular compliance were not observed in the HIV-negative controls. Whilst this may have been because of fewer participants in the HIV-negative arm, the direction and magnitude of the relationships between senescent CD4 cells with cfPWV and crAiX were different in the two groups. This suggests that our findings may be specific to lean PLWH as opposed to lean persons in general. This may explain the observed inverse relationship of CD8+ T-cell activation and CD4+ T-cell senescence and arterial stiffness in PLWH with a low BMI.

The augmentation index is said to be a composite measure of wave reflection and arterial stiffness, and has been suggested to be a more sensitive marker for endothelial function compared to PWV.^27^ The median values of cfAiX and crAiX were more than twice higher in PLWH than the HIV-negative persons. The significant difference observed in the cfAiX by HIV status may indicate that endothelial dysfunction is more pronounced in more elastic vessels as compared to more muscular vessels. Carotid femoral pulse wave velocity is a measure of arterial stiffness in elastic vessels, whereas crPWV in muscular vessels.^26^ A decrease in vascular compliance has been linked to reduced bioavailability of NO, which has several physiological roles, including vasodilation of coronary arteries.^33^ Nitric oxide diffuses through the vascular smooth muscle cells where it activates guanylate cyclase, a process that leads to smooth muscle relaxation. The viral protein Nef has been implicated in the reduction of endothelial NO, an increase in the secretion of cytokines from macrophages and inducing endothelial cell apoptosis.^34^ Using a rat model, Kline, Kleinhenz^18^ reported an association between the HIV proteins and a decrease in vascular and systemic NO bioavailability. This study also reported endothelial dysfunction because of the inability to relax maximally after the injection of acetylcholine. The decrease in NO was reported not to be from eNOS regulation but from the overproduction of superoxide binding to increased NO to form peroxynitrite. However, even though increased in PLWH, our study found no significant difference in 8-isoprostane levels in PLWH.

We observed an inverse association between CD4-activated T cells and 8-isoprostane, suggesting immune activation may not be a driver of increased oxidative stress in lean PLWH. However, the directionality of this relationship could not be assessed in our study. This is in contrast with other studies that reported an increase in immune activation and oxidative stress markers in PLWH as compared to HIV-negative persons.^35^ Mandas, Iorio^36^ reported both an increase in oxidative stress markers and a decrease in antioxidants in the PLWH when compared to the HIV-negative persons.

The cohort of PLWH had been on anti-retroviral therapy for more than 5 years and was assumed to be virally suppressed. However, we observed persistent immune activation of both CD4+ and CD8+ T cells in the PLWH.

With CD4+ and CD8+ T cells being the main components of the immune response, their activation has been associated with disease progression, particularly in those who are treatment naïve.^37,38,39^ Circulating activated immune cells can permeate through to the adventitia and adhere to the endothelium resulting in its dysfunction.^13^ The median values of both activation markers on CD4+ and CD8+ T cells were more than double in PLWH when compared to the HIV-negative persons. We found a significant association between crPWV and activated CD8+ T cells. Paradoxically, this association was inversely related in PLWH and directly related in HIV-negative persons. This draws our attention to the nature of the vasculature being investigated and whether the immune response to elastic versus muscular vessels is different.

The study also saw an inverse association between CD4 senescence markers and cfPWV. Persons living with HIV on treatment or naïve have been described to be immunologically aged when compared to HIV-negative persons, and this has been associated with increased immune activation.^40,41^ This has been attributed to a decrease in the telomere length in DNA molecules.^40,41^ Telomere shortening leads to the initiation of the DNA damage response leading to growth arrest, which if not repaired, leads to permanent growth arrest that is irreversible. At this stage, the cells are neither dead nor functional.

Prior studies of vascular compliance and immune function have mainly been conducted in individuals with higher BMI in cohorts outside of SSA.^9,12,13^ Visceral fat, being metabolically active, is an important site for the secretion of adipokines that influence inflammation, lipid metabolism and insulin sensitivity.^42,43^

Lukich, Gavish^42^ suggested measuring abdominal fat content by waist circumference, as it is a more sensitive measure of endothelial dysfunction than overall body mass measured by BMI. Although our study population was all lean, PLWH had a significantly lower BMI compared to HIV-negative persons. However, waist circumferences were similar between the two groups, potentially reflecting greater visceral obesity in the participants with HIV.^44^

### Strengths and limitations

Our study groups had a far lower median BMI (18.9 kg/m^2^ in the PWLH and 20.7 kg/m^2^ in the HIV-negatives) as compared to similar studies from cohorts in the US and other settings, and we excluded smokers and persons with known cardiovascular pathology. We also assessed a range of T-cell phenotypes and multiple indices of vascular compliance. However, a major limitation of our study is the significantly greater age and small sample size amongst the PLWH compared to HIV-negatives in our study, which precludes attributing the lack of a similar association between T-cell activation and vascular compliance to HIV status. Also, information on the duration of HIV infection before treatment and total CD4 and CD8 T-cell counts were not available. Furthermore, because of the cross-sectional design, causality could not be determined and may not be generalisable as a result of scarcity of information linking these parameters in the African context. Additional longitudinal studies will be needed to explore mechanisms linking immune activation and endothelial dysfunction.^45^

## Conclusion

Lean adults with HIV had increased immune activation and arterial stiffness when compared to HIV-negative persons. Paradoxically, lower arterial stiffness was associated with increasing CD8+ T-cell activation and CD4+ T-cell senescence, in contrast to prior studies in predominantly US cohorts with high proportions of overweight and obese participants. To date, there have been few studies of immune activation and arterial stiffness amongst PLWH in SSA, and further research is needed to determine whether these findings are specific to PLWH with a low BMI or to individuals in SSA in general. Furthermore, the implications of these findings in the context of rising rates of CVD amongst PLWH in the region should be explored further.

## Figures and Tables

**FIGURE 1-A1:**
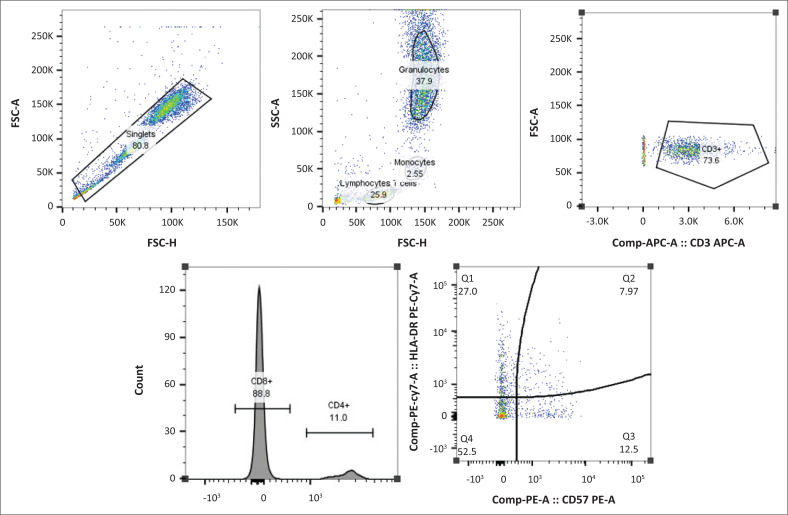
T-cell gating strategy.

## Appendix

**TABLE 1-A1:** Interaction of T-cell subsets and human immunodeficiency virus status with carotid-femoral pulse wave velocity, carotid femoral augmentation index, carotid-radial augmentation index, log-transformed carotid-radial pulse wave velocity and 8-isoprostane.

Outcomes	CD4/CD45RA+ (naïve)			CD4/CD45RA- (memory)			CD4/HLADR-CD57+ (senescent)			CD4/HLADR+CD57- (activated)			CD8/CD45RA+ (naïve)			CD8/CD45RA- (memory)			CD8/HLADR+CD57- (activated)			8- isoprostane		
	β	CI	*P*	β	CI	*P*	β	CI	*P*	β	CI	*P*	β	CI	*P*	β	CI	*P*	β	CI	*P*	β	CI	*P*
cfPWV	0.01	−0.09 – 0.09	0.94	−0.01	−0.09 – 0.09	0.95	−0.22	−0.44 – 0.04	**0.05**	−0.04	−0.25 – 0.17	0.73	0.05	−0.04 – 0.13	0.27	−0.04	−0.12 – 0.05	0.39	−0.12	−0.28 – 0.04	0.13	−0.23	−0.97 – 0.51	0.54
cfAiX	0.79	−0.33 – 1.92	0.16	−0.86	−2.0 – 0.28	0.14	0.42	−2.78 – 3.62	0.79	−0.14	−2.88 – 2.60	0.92	0.14	−1.02 – 1.29	0.81	−0.25	−1.40 – 0.90	0.66	−0.12	−2.29 – 2.04	0.91	8.95	−0.72 – 18.62	0.07
crPWV	−0.01	−0.02 – 0.004	0.24	0.01	−0.004 – 0.02	0.23	−0.01	−0.03 – 0.02	0.93	0.01	−0.02 – 0.03	0.63	0.001	−0.01 – 0.01	0.92	−0.001	−0.01 – 0.01	0.99	−0.01	−0.03 – 0.01	0.32	0.04	−0.04 – 0.11	0.35
crAiX	0.43	−0.81 – 1.67	0.49	−0.56	−1.81 – 0.68	0.37	−2.30	−5.42 – 0.82	0.14	0.32	−2.33 – 2.97	0.81	−0.30	−1.51 – 0.90	0.61	0.18	−1.02 – 1.38	0.76	0.49	−1.61 – 2.60	0.64	5.35	−5.12 – 15.82	0.31
8-isoprostane	0.09	−0.002 – 0.18	**0.05**	−0.09	−0.18 – 0.002	0.06	0.05	−0.24 – 0.34	0.72	0.02	−0.24 – 0.28	0.89	0.04	−0.04 – 0.13	0.32	−0.04	−0.13 – 0.04	0.32	−0.10	−0.28 – 0.08	0.27	-	-	-

Note: Bold indicates statistically significant value.

CI, confidence interval; cfPWV, carotid-femoral pulse wave velocity; crPWV, carotid-radial pulse wave velocity; cfAiX, carotid-femoral augmentation index; crAiX, carotid-radial augmentation index.

**TABLE 2-A2:** Relationship of T-cell subsets with carotid-femoral pulse wave velocity, carotid-femoral augmentation index, carotid-radial augmentation index and log-transformed carotid radial pulse wave velocity in human immunodeficiency virus negative participants.

Outcomes	CD4/CD45RA+ (naïve)			CD4/CD45RA- (memory)			CD4/HLADR-CD57+ (senescent)			CD4/HLADR+CD57- (activated)			CD8/CD45RA+ (naïve)			CD8/CD45RA- (memory)			CD8/HLADR+CD57- (activated)			8- isoprostane		
	β	CI	*P*	β	CI	*P*	β	CI	*P*	β	CI	*P*	β	CI	*P*	β	CI	*P*	β	CI	*P*	β	CI	*P*
cfPWV	0.02	−0.04 – 0.08	0.52	−0.02	−0.08 – 0.05	0.59	0.01	−0.16 – 0.17	0.93	0.04	−0.12 – 0.20	0.60	−0.01	−0.07 – 0.05	0.76	0.001	−0.06 – 0.06	1.0	0.05	−0.74 – 0.18	0.40	0.15	−0.33 – 0.62	0.52
cfAiX	−0.94	−2.18 – 0.29	0.13	0.98	−0.29 – 2.26	0.12	0.29	−3.00 – 3.57	0.86	0.36	−2.89 – 3.60	0.82	−0.17	−1.37 – 1.03	0.77	0.27	−0.92 – 1.45	0.64	0.41	−2.19 – 3.02	0.75	−12.49	−20.33 – 4.64	**<0.01**
crPWV	0.01	−0.004 – 0.02	0.21	−0.01	−0.02 – 0.004	0.24	−0.01	−0.02 – 0.02	0.57	0.01	−0.03 – 0.02	0.80	0.01	−0.01–0.01	0.62	−0.01	−0.01 – 0.01	0.58	0.01	−0.01 – 0.03	0.55	−0.04	−0.10 – 0.03	0.26
crAiX	−0.26	−1.39 – 0.87	0.64	0.35	−0.81 – 1.51	0.54	1.22	−1.58 – 4.01	0.38	0.18	−2.63 – 3.0	0.89	−0.15	−1.19 – 0.89	0.77	0.30	−0.73 – 1.32	0.55	0.21	−2.05 – 2.47	0.85	−5.41	−15.30 – 4.49	0.26

Note: Bold indicates statistically significant value.

CI, confidence interval; cfPWV, carotid-femoral pulse wave velocity; crPWV, carotid-radial pulse wave velocity; cfAiX, carotid-femoral augmentation index; crAiX, carotid-radial augmentation index.

**TABLE 3-A3:** Analysis of variance table for the interaction effect of CD4 senescent cells and human immunodeficiency virus on carotid-femoral pulse wave velocity.

Variable	Degrees of freedom	*F*	*P*
Age	1	1.66	0.21
BMI	1	0.99	0.33
HIV status	1	0.20	0.66
CD4 senescent cells	1	0.00	0.99
HIV status: CD4 senescent cells interaction	1	4.21	0.05
Regression	5	2.65	0.04
**Total**	**51**	**-**	**-**

BMI, body mass index; HIV, human immunodeficiency virus; CD4, cluster of differentiation 4.

**TABLE 4-A4:** Analysis of variance table for the interaction effect of senescent CD4 T cells and human immunodeficiency virus on carotid-radial augmentation index.

Variable	Degrees of freedom	*F*	*P*
Age	1	3.22	0.08
BMI	1	1.93	0.17
HIV status	1	3.23	0.08
CD4 senescent cells	1	1.03	0.32
HIV status: CD4 senescent cells interaction	1	4.81	0.03
Regression	5	3.23	0.01
**Total**	**51**	**-**	**-**

BMI, body mass index; HIV, human immunodeficiency virus; CD4, cluster of differentiation 4.
